# Catalytic nanozyme Zn/Cl-doped carbon quantum dots as ratiometric fluorescent probe for sequential on-off-on detection of riboflavin, Cu^2+^ and thiamine

**DOI:** 10.1038/s41598-022-23055-6

**Published:** 2022-10-31

**Authors:** Sahar Dadkhah, Ali Mehdinia, Ali Jabbari, Ahmad Manbohi

**Affiliations:** 1 Department of Chemistry, Faculty of Science, K. N., Toosi University of Technology, Tehran, Iran; 2 Iranian National Institute for Oceanography and Atmospheric Science, Tehran, Iran

**Keywords:** Bioanalytical chemistry, Fluorescent probes, Sensors

## Abstract

A novel metal-doped Zn/Cl carbon quantum dots (Zn/Cl-CQDs) was developed successfully as ratiometric fluorescent probes for the sequential on-off-on detection of riboflavin, Cu^2+^ ion and thiamine. The excellent catalytic performance of the Zn/Cl-CQDs nanozyme serves as an ideal platform for sensitive detection of thiamine. Due to the addition of riboflavin to the Zn/Cl-CQDs, the blue emission peak of Zn/Cl-CQDs at 440 nm remains unaffected and used as an internal reference approach, while the green emission peak of riboflavin at 520 nm appeared and increased remarkably. Following the presence of Cu^2+^, a quenching blue fluorescence signal of Zn/Cl-CQDs was observed which resulted in consequent fluorescent ‘turn-off’ response toward Cu^2+^ ion. Finally, upon the addition of thiamine to the above solution under alkaline condition, the blue emission of Zn/Cl-CQDs was gradually recovered. The prepared Zn/Cl-CQDs could act as a nanozyme catalyst for directly catalyzing the oxidation of non-fluorescent substrate of thiamine to produce highly fluorescent substrate of thiochrome. As a result, the blue fluorescence emission peak at 440 nm was recovered. Eventually, the sequential detection properties of ratiometric probes for riboflavin, Cu^2+^ ion and thiamine were successfully applied in VB2 tablets, drinking water and VB1 tablet with good recoveries of 96.21%, 98.25% and 98.44%, respectively.

## Introduction

In recent years, development of metal-doped carbon quantum dots (CQDs) has attracted much attention and become an effective strategy to adjust their surface chemical reactivities and electronic properties in many applications from separation science and detection of analytes^1,2^. Up to now, some articles have been reported about catalytic performance of metal-doped carbon quantum dots^3^. Duan et al. have developed Cu-doped CQDs (Cu-CQDs) as the catalyst for chemiluminescence detection of glucose^4^. Iron and nitrogen co-doped carbon dots (Fe-N-CQDs) were prepared by Yang’s group. The nanozyme, Fe-N-CQDs exhibited a superior peroxidase activity due to the iron doping and were successfully applied in immunosorbent assay^5^.

The measurements with single emission fluorescence probe can be affected by different external factors such as light scattering of the sample matrix, temperature fluctuation, excitation intensity drift, variation in the probe concentration, environment and solvent system. To overcome these undesirable effects, the construction of ratiometric fluorescent probes as an excellent candidate for biochemical/chemical sensing platform has attracted wide attention^6^. Contrary to conventional methods of single emission fluorescence for biochemical/chemical detection, the ratiometric fluorescence methods include an intrinsic built-in self-calibration for correction of interferences arisen from background and sample matrix. In ratiometric fluorescence probes, fluorescence intensity of the analytes changes from two or multi-emission bands at various wavelengths, which can provide reliable and accurate quantitative analysis^7,8^.

In recent years, carbon dots have presented convenient platforms to design and construct ratiometric fluorescent probes. In ratiometric fluorescent probes based on CQDs, there are two major strategies for constructing dual-emission ratiometric fluorescent probes. One is to apply the CQDs as a reference signal which does not respond to the analyte. The other is to introduce CQDs as response signals that are sensitive to analyte and respond to the analyte under optimal conditions^7,9,10^. In many literatures based on CQDs ratiometric fluorescent probes, the CQDs were used as either analyte-response^11,12^ or reference unit^13,14^. Typically, these CQDs-based ratiometric fluorescent probes have been extended with a series of nanohybrid of CQDs with other organic dyes such as Rhodamine^15^, metal organic framework (MOF)^16^, nanoclusters (AuNCs and CuNCs)^17,18^ and various QDs (carbon-based quantum dots, semiconductor quantum dots CdTe^19^ and CdSe^20^) for different analytes. All results from the articles confirmed that the target analytes were determined with good selectivity, high accuracy and reproducibility, based on the self-calibration and background correction due to the fluorescence intensity ratio of emission peaks from the response and reference signals.

However, the construction of the sequential multi-component detection of analytes based on ratiometric fluorescent probes is a major challenge because it requires two or more fluorophore groups to be excited by the same excitation wavelength. Additionally, the construction of ratiometric fluorescent probes is not straightforward and does not involve a simple mixing procedure. Due to the importance of simultaneous detection of multiple analytes in different matrices, synthesis and design of biochemical/chemical probes for sequential multi-component detection of analytes have recently drawn great attention^9,16,21,22^. Generally, these sensors could respond to multiple ions^23^ or small molecules^24^, even some of them can sequentially detect ions or molecules^25^.

Copper ion (Cu^2+^), Riboflavin (Vitamin B_2_) and thiamine (Vitamin B_1_) are three components that are quite related to our environment and lives. Riboflavin, commonly referred to vitamin B_2_, is an important water-soluble vitamin and plays an essential role in the normal functioning of the body by participating in the normal metabolism of proteins, carbohydrates, fats and cellular processes. Riboflavin cannot be generated by the human body, and it can merely be obtained from food or pharmaceutical supplements^26–28^.

Copper is the most abundant metal in the human body, and has an essential role in ferroxidase, cytochrome oxidase and superoxide dismutase as a cofactor enzyme. This essential element can help to produce red blood cells and support transcriptional events in human body. However, the accumulation of excess amounts of copper Cu^2+^ can interrupt the balance between cells, which leads to a category of illnesses such as Menkes, Wilson, Parkinson’s and Alzheimer’s diseases. The accumulation of excess amounts of Cu^2+^ in the body leads to gastrointestinal disorders and liver or kidney disorders^19,29,30^. Cu^2+^ can also cause environmental pollution due to its widespread use in agriculture and industry. Hence, according to the WHO guidelines, the maximum allowable level of Cu^2+^ in drinking water is 2 mg L^−1^ (32 μM). Therefore, it is very important to develop an efficient and accurate method to detect Cu^2+^ ion, particularly in drinking water monitoring^31^.

Thiamine, a sulfur-containing water-soluble vitamin and natural nutrient is present in many foods. Thiamine has an essential role in the metabolism of enzymes, brain health especially in emotional well-being, concentration and focus. Thiamine deficiency causes various illnesses in the human body such as beriberi, nervous and cardiovascular problems. Since this compound is only synthesized by bacteria, fungi and plants, adequate dietary intakes are necessary. Therefore, the development of an accurate, convenient and sensitive method for determination of vitamin B_1_ in food and pharmaceutical supplements is crucial^32,33^.

As far as we know, this is the first study to offer insights into the implementation of proper analytes sequence as part of response or reference units in ratiometric fluorescent probe develpment. Moreover, superior catalyst activity of Zn/Cl-CQDs as a nanozyme for directly catalyzing the oxidation of thiamine were demonstrated.

In this study, proper selection of analytes, their rational combinations and sequence detection under optimal condition for design and construction of new ratiometric fluorescent probes were evaluated. In other words, the addition of the first analytes to the CQDs probe can act as a reference or response signal for the detection of subsequent analytes. As a result, we aimed to design a ratiometric fluorescent probe based on CQDs for sequential monitoring of riboflavin, Cu^2+^ ion and thiamine in real samples, which enriches the range of CQDs applications within various fields of chemical and biological assays.

## Experimental

### Materials and instruments

Thiamine hydrochloride, Pyridoxine hydrochloride (vitamin B6), calcium D-pantothenate (vitamin B5), ascorbic acid (vitamin C), NaOH, NaCl, KCl, ZnCl_2_, PbCl_2_, FeCl_2_, MnCl_2_, CrCl_2_, CdCl_2_, CoCl_2_, MgCl_2_, CaCl_2_ were bought from Merck (Darmstadt, Germany). Riboflavin, niacin (vitamin B3) and zinc gluconate were purchased from Sigma-Aldrich.

For all spectroscopic and experimental studies, Milli-Q water (0.05 μScm^−1^) was used. Fluorescence spectra were obtained on LS-55 Perkin Elmer fluorescence spectrophotometer from 370 to 800 nm. The IR spectra of Zn/Cl-CQDs was performed on FT-IR Spectrum two model Perkin Elmer from 400 to 4000 cm^−1^.The electron microscopy of Zn/Cl-CQDs was conducted using a SEM (ZEISS with EDS and Mapping) and high-resolution FEI Tecnai F20 TEM.

### Synthesis of Zn/Cl-doped CQDs by microwave-assisted method

A novel carbon quantum dots co-doped with metal and non-metal elementals, zinc and chlorine, (Zn/Cl-CQDs) was prepared by one-pot microwave-assisted method with zinc gluconate and hydrochloric acid. Briefly, 1 g of zinc gluconate was firstly weighed and added to 10 mL of ultrapure water and stirred for 10 min. Then, the pH of the mixture was adjusted to about 4 using HCl solution. The solution was then placed in the microwave radiation system at temperature 160 °C for 10 min. Finally, the color of the solution turned to orange, which confirmed the formation of Zn/Cl-CQDs. Then the pH of the final solution was neutralized and the Zn/Cl-CQDs solution was stored in 4 °C for further use.

### Detection procedure for sequential fluorescent detection

Initially, stock solutions of riboflavin, Cu^2+^ ion and thiamine were prepared in ultrapure water at a high concentration and further diluted. The Zn/Cl-CQDs nanosensor was utilized for sequential detection of riboflavin, Cu^2+^ ion and thiamine.

1 mL of Zn/Cl-CQDs nanosensor and 3.8 mL of ultrapure water were added into a 5 mL test tube. Then, 200 μL of riboflavin working solutions with various concentrations were added into above solution. Then the fluorescence spectra of the solution were measured from 370 to 800 nm by fluorescence spectrophotometer at excitation wavelength 366 nm. Under optimal condition, Zn/Cl-CQDs and riboflavin system were used for sequential detection of Cu^2+^ ion. 1 mL of Zn/Cl-CQDs solution and 3.6 mL of ultrapure water, 200 μL of riboflavin (500 nM) were added into a 5 mL tube. Then, 200 μL of Cu^2+^ ion working solutions with various concentrations were added into above solution and the fluorescence intensities of the mixture solution were measured from 370 to 800 nm. Finally, the quantitative detection of thiamine using above system was performed as follows: 1 mL of Zn/Cl-CQDs solution, 200 μL of riboflavin, 200 μL of Cu^2+^ ion (160 µM) and 200 μL of thiamine with different concentrations, 800 μL NaOH (0.05 mM) and 2.6 mL of ultrapure water were added into a 5 mL tube. The fluorescence intensities of the mixture solution were measured from 370 to 800 nm. To evaluate the linearity and limit of detection for the sequential detection of riboflavin, Cu^2+^ ion and thiamine, the spectra of fluorescence intensities of the Zn/Cl-CQDs with different concentrations of the analytes were recorded.

### Real samples preparation

VB_2_ (riboflavin) and VB_1_ (thiamine) tablets were purchased for real samples evaluation. Tablets were ground into powder and dissolved in 100 mL water. The solution was centrifuged at 10,000 rpm for 10 min to remove the undissolved substances. Then the upper solution was filtered through a 0.45 μm filter membrane. The sample solution was further diluted in order to prepare appropriate concentrations of analytes for the quantitative analysis. Bottles of drinking water were purchased from supermarket and analyzed without any further pre-treatment.

## Results and discussion

### Characterization of Zn/Cl-CQDs

**Figure 1:**
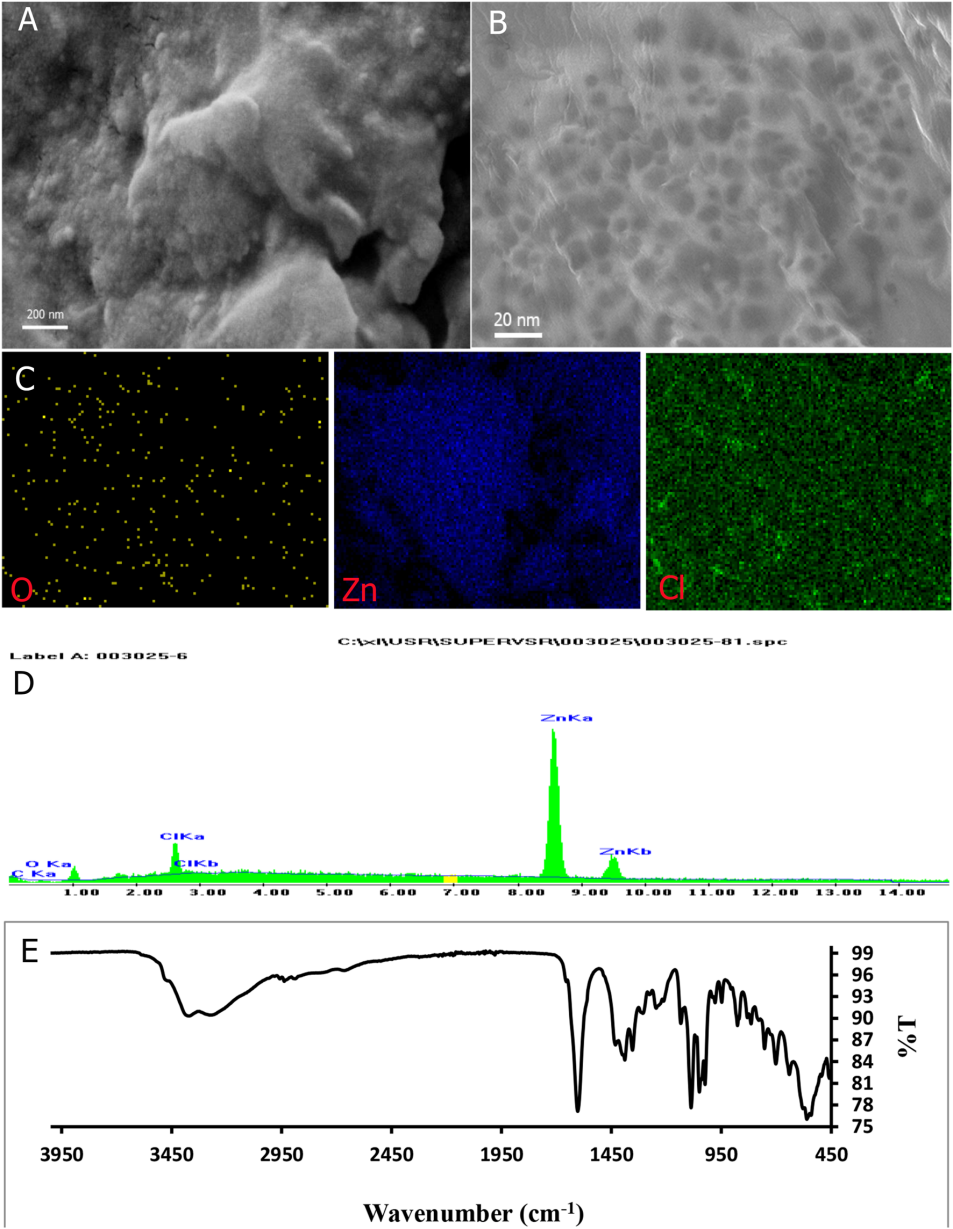
(**A**) FE-SEM (**B**) HR-TEM. (**C,D**) EDX elemental mapping and (**E**) FT-IR spectra of Zn/Cl-CQDs.

The morphology and elemental analysis of Zn/Cl-CQDs were performed by the SEM instrument equipped with EDX spectroscope. Figure 1A reveals spherical morphology of the Zn/Cl-CQDs. Moreover, EDX analysis and mapping exhibited the exact composition and distribution of doped elements in details. As shown in Fig. 1D, EDX mapping, the uniform and successful doping of Zn and Cl elements in the structure of Zn/Cl-CQDs were confirmed. In addition, Fig. 1C indicates that C, O, Zn and Cl elements were homogeneously distributed throughout the structure of synthesized Zn/Cl-CQDs. As a result, high efficiency of elemental doping through microwave assisted synthesis strategy, led to uniform distribution of elements in the structure of Zn/Cl-CQDs. The morphology and structure of Zn/Cl-CQDs are shown in Fig. 1B. The TEM image shows that the average size distribution of Zn/Cl-CQDs is mainly smaller than 20 nm. On the other hand, analysis of TEM image by ImageJ software showed the distribution size of 0–17 nm with the average of about 9 nm.

The surface structure and composition of Zn/Cl-CQDs were further evaluated by FT-IR (Fig. 1E). In the FT-IR spectra of the Zn/Cl-CQDs, a broad absorption band at about 3000–3500 cm^−1^could be attributed to the stretching vibration of O–H groups^4^. Moreover, the peaks at 1220 cm^−1^, 1022 cm^−1^ and 1133 cm^−1^ were assigned to the asymmetric and symmetric stretching vibrations of C − O and the stretching vibration of the C − O − C, respectively. The FT-IR spectra of Zn/Cl-CQDs also showed absorption peaks at around 2936 cm^−1^ and 2887 cm^−1^ which were related to stretching and bending vibrations of methylene (− CH_2_) groups^34^. It should be noted thatthe important functional bands at 753 cm^−1^ and 561 cm^−1^were related to the stretching vibrations of Zn–O and C–Cl bands, indicating the successful doping of zinc and chlorine elements in the CQDs^35,36^. The FT-IR results were in agreement with other characterization results and proved that Zn/Cl-CQDs mainly contained hydroxyl, carbonyl, carboxylic acid, epoxy groups on its surface and also indicated that Cl and Zn heteroatoms were successfully doped.

### Sequential detection of riboflavin, Cu^2+^ ion and thiamine based on Zn/Cl-CQDs

**Figure 2:**
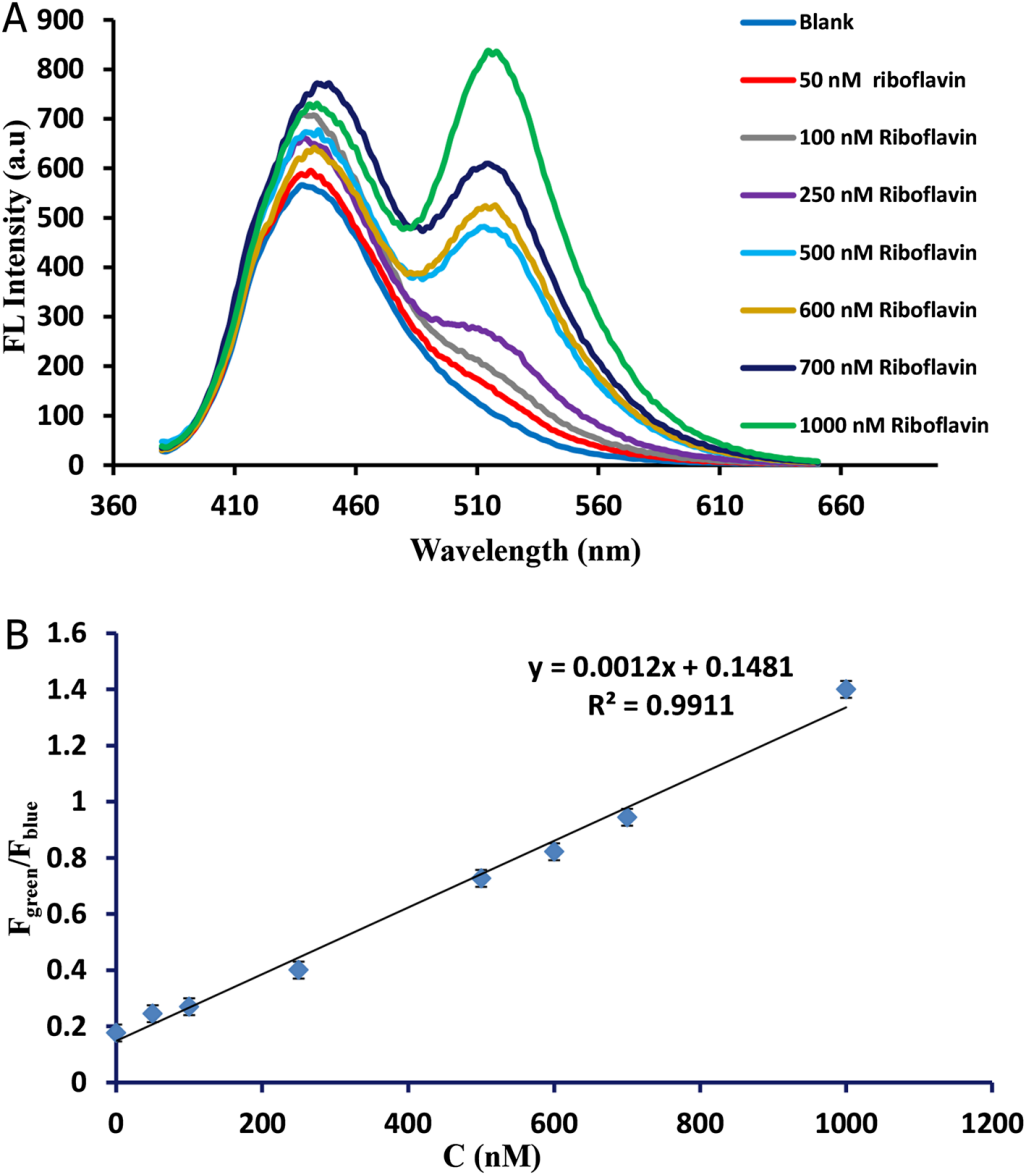
(**A**) The ratio of the relative green to blue emission peaks (F_green_/F_blue_) upon successive addition of riboflavin. (**B**) Linear regression graph of F_green_/F_blue_ values against riboflavin concentration in the ranges of 50–1000 nM.

The potential of Zn/Cl-CQDs as a new ratiometric fluorescent probe for monitoring of riboflavin was evaluated. As shown in Fig. 2A, a new green emission peak at 520 nm appeared and increased remarkably by the gradual addition of riboflavin to the Zn/Cl-CQDs solution; while the blue emission peak of Zn/Cl-CQDs at 440 nm remained unaffected or was accompanied by very small changes. In the designed sensors for detection of riboflavin, the Zn/Cl-CQDs could be served as an internal reference unit that is target insensitive, while a new emission peak of riboflavin at 520 nm appeared and increased. A suitable linear relation between the ratio of the relative fluorescence intensities of green to blue emission peaks (F_green_/F_blue_) with concentration of riboflavin was obtained in the range of 50–1000 nM (Fig. 2B). The equation of calibration curve was (F_green_/F_blue_) = 0.0012x + 0.1481 with a good correlation coefficient (0.9911) and limit of detection (14.12 nM).

**Figure 3:**
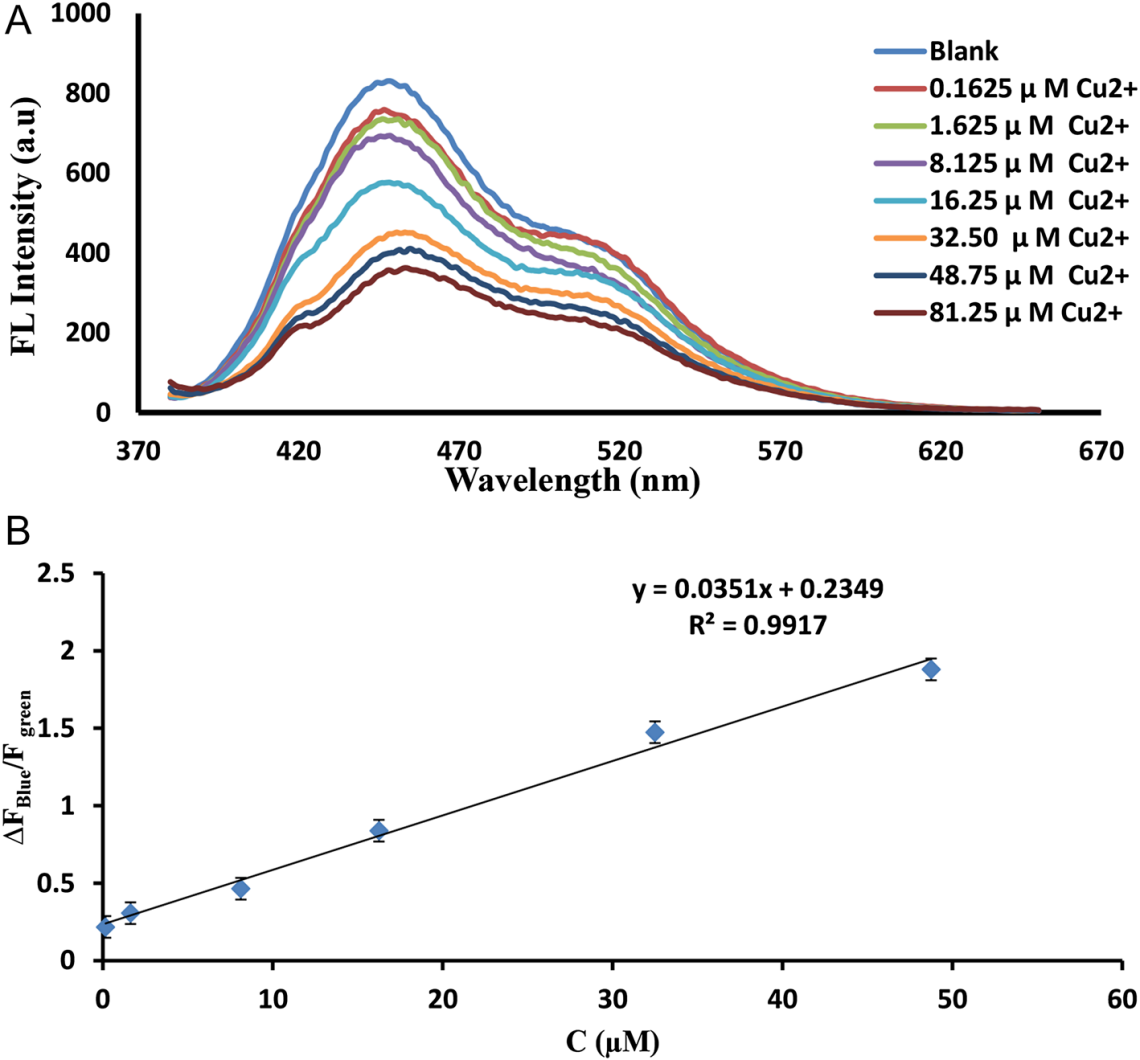
(**A**) The ratio of the fluorescence signal of blue wavelength to the green peaks (∆F_Blue_/F_green_) upon successive addition of Cu^2+^ ion. (**B**) Linear regression graph of (∆F_Blue_/F_green_) values against Cu^2+^ ion concentration in the ranges of 0.162–48.75 µM.

Then sequential detection of Cu^2+^ ion using the Zn/Cl-CQDs /riboflavin system was investigated. As displayed in Fig. 3A, when the concentration of Cu^2+^ ion in the Zn/Cl-CQDs/riboflavin solution increased, the fluorescence signal at blue and green wavelengths gradually decreased. The difference was that the decrease in fluorescence signal occurred more intensely in the blue wavelength than green. A suitable linear relation was obtained between the ratio of the fluorescence intensities of blue wavelength to the green peaks (∆F_Blue_/F_green_) and concentration of Cu^2+^ ion in the range of 0.162–48.75 µM (Fig. 3B). The equation of the calibration curve was (∆F_Blue_/F_green_) = 0.0351x + 0.2349 with a good correlation coefficient (0.9917) and limit of detection (0.0543 µM).

**Figure 4:**
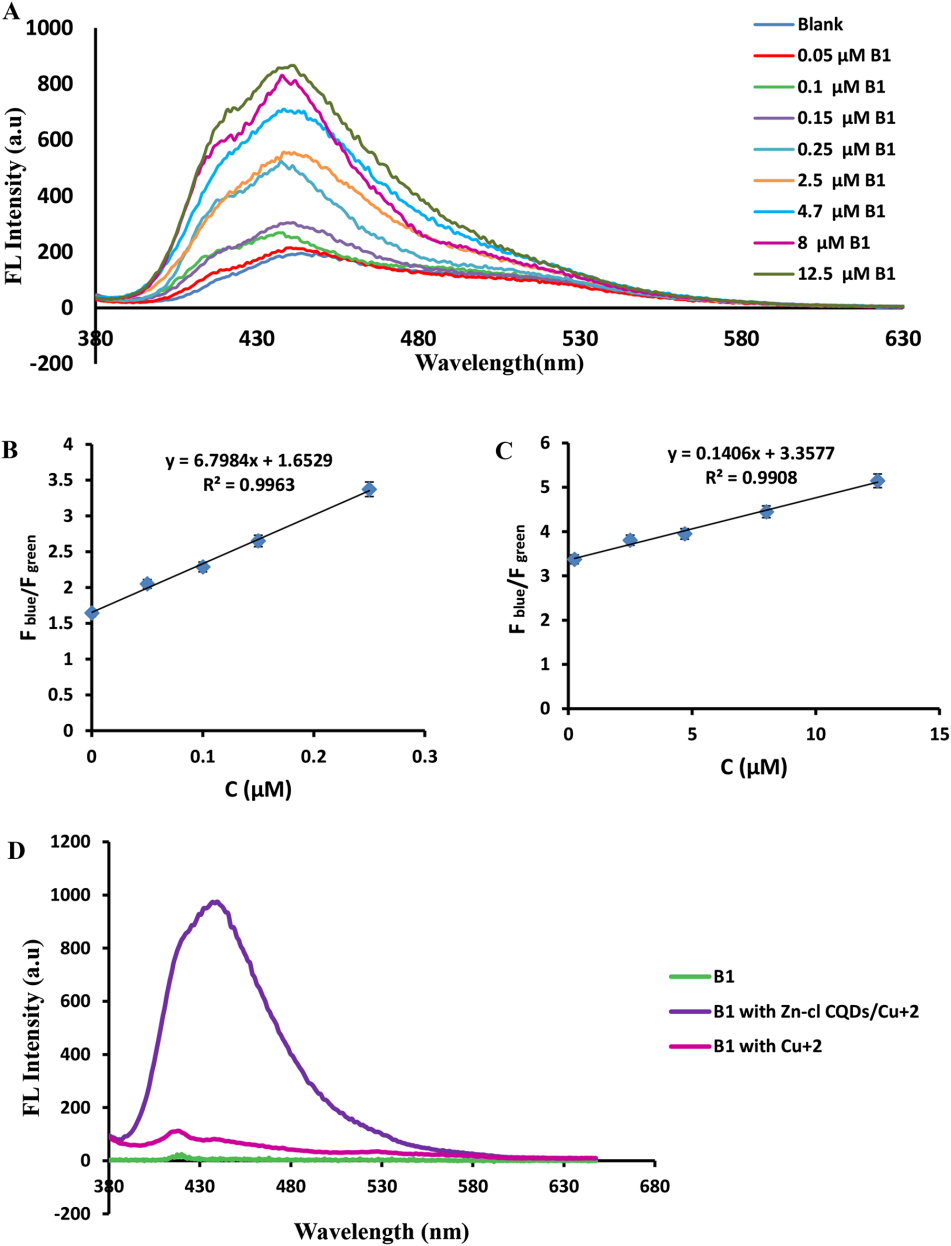
(**A**) The ratio of the relative fluorescence signal of blue to green emission peaks (F_blue_/F_green_) upon successive addition of thiamine. (**B,C**) Linear regression graph of F_blue_/F_green_ values against thiamine concentration in two linear ranges of 0.05–0.25 μM and 0.25–12.5 μM. (**D**) The fluorescence spectra of pure thiamine in the presence and absence of Cu^2+^ ion and Zn/Cl-CQDs.

Thiamine as the third analyte was selected to prove sequential detection performance of the designed probes. Finally, increasing the concentration of thiamine to the above probe solution in alkaline conditions and in the presence of copper resulted in recovery of the blue fluorescence emission corresponding to CQDs, while the change in fluorescence signal of the green wavelength was negligible (Fig. 4A). There were two linear ranges from 0.05–0.25 μM and 0.25–12.5 μM for thiamine (Fig. 4B,C) related to the linear equations of F_blue_/F_green_ = 6.7984 C + 1.6529 (*R*^2^ = 0.9963) and F_blue_/F_green_ = 0.1406 C + 3.3577 (*R*^2^ = 0.9908), respectively.

A comprehensive comparison of the fluorescent probes for the detection of riboflavin, Cu^2+^ ion and thiamine were also performed and listed in Table 1. The sequential multi-component detection of analytes based on new Zn/Cl-CQDs probes exhibited high sensitivity, good linear range and low limit of detection which was better than the compared resources (Table 1).

**Table 1:** Comparison of different fluorescence-based probes for the detection of riboflavin, Cu^2+^ and thiamine.

FL Probe	Analytes	Linear range	Detection limit	References
NPCDs	Riboflavin	0.5–50 µM	0.17 µM	^26^
g-CNQDs@Zn-MOF	Riboflavin	0.005–1 µM	15 nM	^27^
N, S-CDs	Riboflavin	0.56–7.44 µM	1.9 nM	^28^
C-dot@SiO_2_@Q-dots	Cu^2+^	0.1–1 µM	0.096 µM	^19^
GG-Dns	Cu^2+^	–	0.29 µM	^29^
Ri-AuNCs	Cu^2+^	0–30 µM	0.9 µM	^31^
Arg-GQDs	Thiamine	53 nM	0.1–8 µM	^32^
e-PNPs	Thiamine	0.1–25 µM	2.6 nM	^33^
Zn/Cl-CQDs	Riboflavin	50–1000 nM	14.12 nM	This work
	Cu^2+^	0.162–81.25 µM	0.0543 µM	
	Thiamine	0.05–12.5 µM	0.0103 µM	

*NPCDs* Nitrogen and phosphorus co-doped carbon dots, *g-CNQDs@Zn- MOF* Graphitic carbon nitrides quantum dots-Zn-MOF composite, *N, S-CDs* N, S doped carbon dots, *C-dot@SiO2@Q-dots* Silica-coated carbon dots conjugated to CdTe quantum dots, *GG-Dns* Dansyl-based fluorescent probe bearing a glycylglycine group, *Ri-AuNCs* Riboflavin-stabilized gold nanoclusters, *Arg-GQDs* Arginine-functionalized graphene quantum dots, *e-PNPs* Exhibiting polymer nanoparticles.

In order to investigate the practical nanozyme catalyst activity of the introduced Zn/Cl-CQDs, the fluorescence spectra of thiamine in the absence and presence of Zn/Cl-CQDs were recorded and exhibited in Fig. 4D. In the present investigation, it was found that a non-fluorescent substrate of thiamine could be oxidized into a fluorescent substrate of thiochrome by copper ion in the presence of Zn/Cl-CQDs as catalyst. As shown in Fig. 4D, this phenomenon was not observed in the absence of Cu^2+^ ion and Zn/Cl-CQDs. The results proved that the design of Zn/Cl-CQDs and proper selection of analytes (Cu^2+^ ion and thiamine) and their rational combinations under optimal condition could lead to construction of desirable sequential multi-component detection of analytes based on on–off-on strategy of ratiometric probes.

### Wavelengths selection for ratiometric fluorescence detection

**Figure 5:**
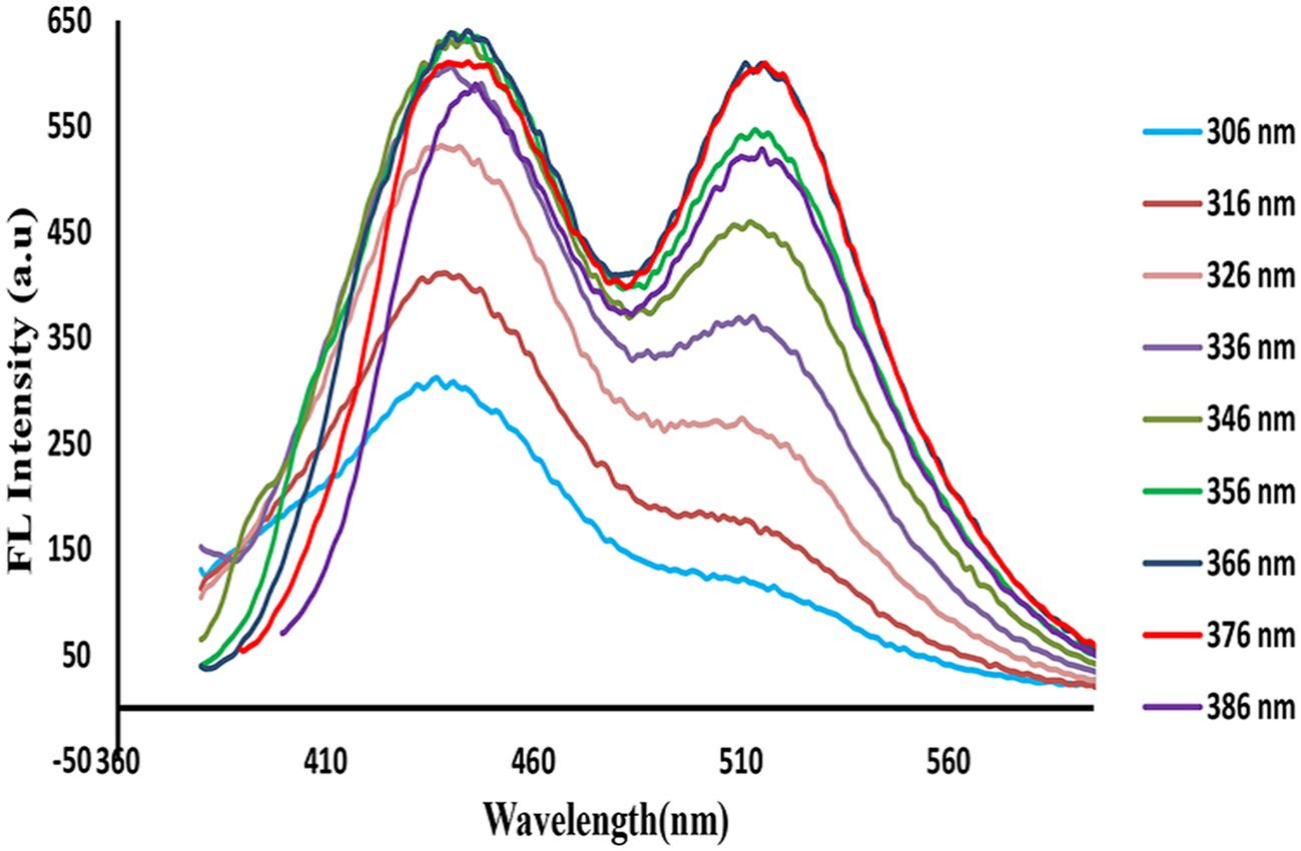
The emission spectra of Zn/Cl-CQDs /riboflavin system under various excitation wavelength from 306 to 386 nm.

The excitation-dependent fluorescence emission features of the Zn/Cl-CQDs/Riboflavin were studied by fluorescence spectroscopy. As exhibited in Fig. 5, Zn/Cl-CQDs /riboflavin solution consisted of two emission peaks observed at 440 nm and 520 nm under various excitation wavelengths from 306 to 386 nm. It was clearly shown that the intensities of the emission peaks at 440 and 520 nm gradually increased with changing the excitation wavelength from 306 to 366 nm. Then, by increasing the excitation wavelength from 366 to 386 nm, the fluorescence intensities of both wavelengths related to carbon dot and riboflavin decreased in 440 nm and 520 nm, respectively. Therefore, the maximum fluorescence emission intensities of both Zn/Cl-CQDs and Riboflavin were observed at excitation wavelength of 366 nm. Moreover, 366 nm was selected as the optimal excitation wavelength.The results in Fig. 5 demonstrated that the Zn/Cl-CQDs /riboflavin had a great potential for designing dual emission ratiometric fluorescent probes, which could be achieved with an optimum single excitation wavelength (366 nm) for the subsequent detection of analytes.

### Mechanism description

#### Riboflavin

In the designed sensors for detection of riboflavin, the blue emission peak of Zn/Cl-CQDs at 440 nm remainen unaffected and was used as an internal reference approach, while riboflavin was directly detected by measuring its own emission at 520 nm. This ratiometric fluorescence method for the detection of riboflavin led to self-calibration for correction of interferences arisen from background and detection systems, which was based on the changes in the ratio of two emission peaks of response/reference units (F_green_/F_blue_).

#### Cu^2+^

Then sequential detection of Cu^2+^ ion using the Zn/Cl-CQDs /riboflavin system was investigated. As displayed in Fig. 3A, when the concentration of Cu^2+^ion increased,the fluorescence signal gradually decreased. Cu^2+^ is an electron acceptor and exhibits a strong affinity with the oxygen group^37^. The quenching mechanism could be mainly due to a photoinduced electron transfer (PET) process from excited C-dots to the empty d orbits of Cu^2+^^38^. Also, investigation on the surface properties of Zn/Cl-CQDs by the FT-IR revealed the presence of hydroxyl and carboxyl group of CQDs, suggested the possible preferential probability of coordination with Cu^2+^ and quenching fluorescence by an electron transfer mechanism.

#### Thiamine

The catalytic performances of metal-doped carbon quantum only could be found in a few studies^39,40^. Zinc, an important element assisting the electron-transfer process could be a promising metal dopant for CQDs, which indicated catalytic performances due to the higher oxidation state of Zn in the whole CQDs structures^40^. The possible mechanism for detection of thiamine can be explained by this fact that the non-fluorescent substrate of thiamine could be oxidized to a blue fluorescent product of thiochrome in the presence of catalyst or oxidizing agent in alkaline condition^41^.

In these studies, the catalytic activities of the Zn/Cl-CQDs were evaluated by the quantification of oxidation product of thiamin named as thiochrome with fluorescence properties and detected via fluorescence spectroscopy. To investigate the practical nanozyme catalyst activity of the introduced Zn/Cl-CQDs, the fluorescence spectra of thiamine in the absence and presence of Zn/Cl-CQDs were recorded and exhibited in Fig. 4D.

Finally, upon the addition of thiamine to the above solution under alkaline condition resulted in the gradual recovery of Zn/Cl-CQDs blue emission. The prepared Zn/Cl-CQDs could act as a nanozyme catalyst for directly catalyzing the oxidation of non-fluorescent substrate of thiamine to produce highly fluorescent substrate of thiochrome, thus the blue fluorescence emission peak at 440 nm was recovered.

### Interference and selectivity studies

**Figure 6:**
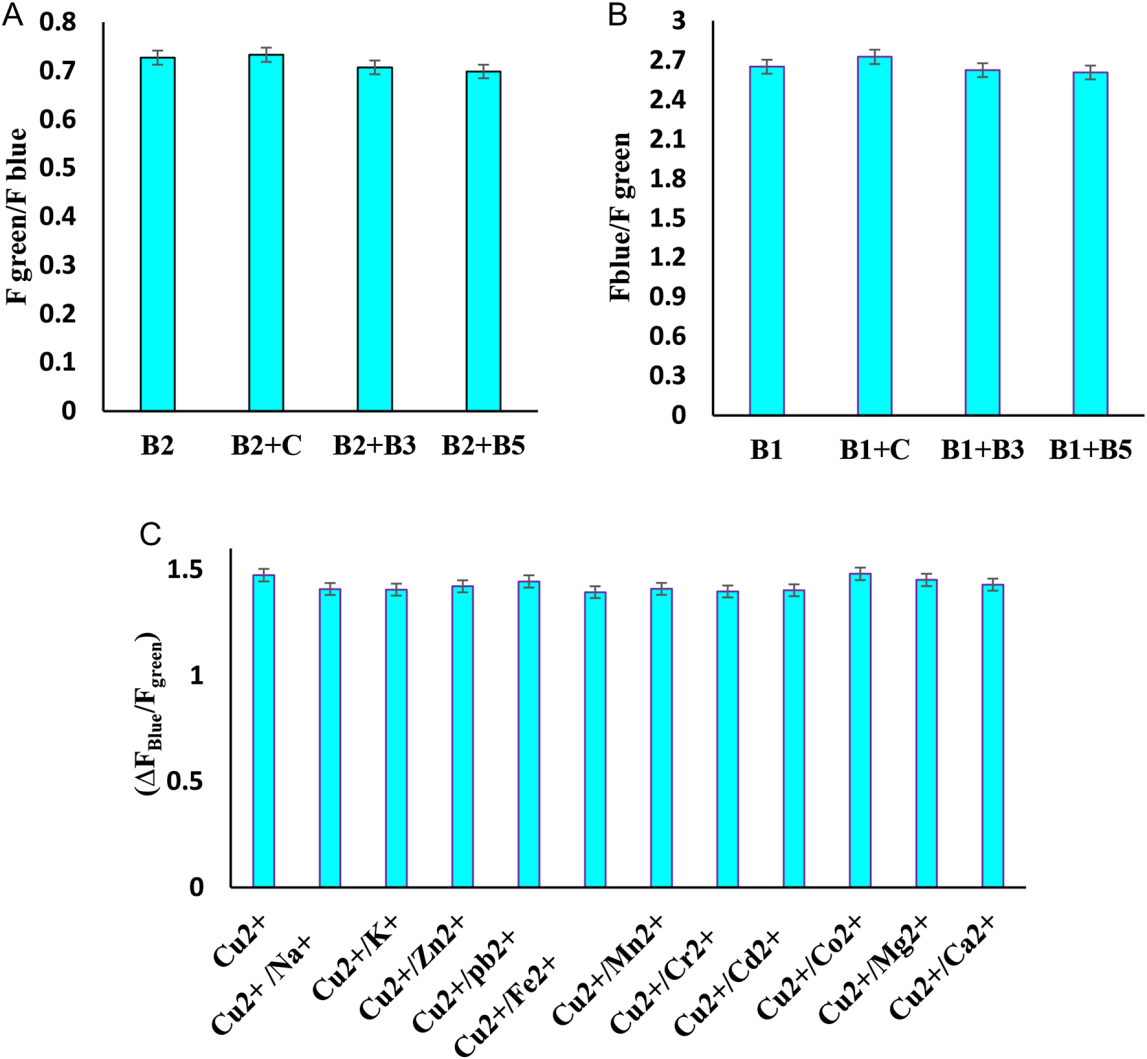
Selectivity studies of the Zn/Cl-CQDs nanosensor (**A**) The fluorescence intensity of the Zn/Cl-CQDs sensing system for riboflavin (500 nM) in the presence and absence of different interfere Vitamins C, B3, B5 with concentration of 5000 nM. (**B**) The fluorescence intensity of the Zn/Cl-CQDs sensing system for thiamine (0.16 µM) in the absence and presence of different interfere other vitamins (C, B3, B5) with concentration of 1.6 µM. (**C**) Change in fluorescence intensity of the Zn/Cl-CQDs sensing system for Cu^2+^ (32.5 µM) in the absence and presence of different interfere ions (including Na^+^, K^+^, Zn^2+^, Pb^2+^, Fe^2+^, Mn^2+^, Cr^2+^, Cd^2+^, Co^2+^, Mg^2+^, Ca^2+^) with concentration of 325 µM.

The effect of different common substances used in the formulation of supplements was evaluated as interfering factor in accurate determination of riboflavin. Vitamin C, B_3_, B_5_ with concentration of 5000 nM were used as competitive water-soluble vitamins for selectivity studies of the probes for riboflavin (500 nM). As shown in Fig. 6A, no change or a slight change (< 4%) in the fluorescence signal of sensor (F_green_/F_blue)_ was observed for riboflavin monitoring in the presence of other vitamins. The results confirmed that, even at concentrations 10 times that of the interfering vitamins, it has little effect on the riboflavin detection.

In order to examine the selectivity of the probes for Cu^2+^ ion, the fluorescence response of Zn/Cl-CQDs /riboflavin solution probe was verified in the presence of different metal ions (including Na^+^, K^+^, Zn^2+^, Pb^2+^, Fe^2+^, Mn^2+^, Cr^2+^, Cd^2+^, Co^2+^, Mg^2+^, Ca^2+^). As shown in Fig. 6C, the addition of these metal ions with concentration of 325 µM caused no apparent changes (< 6%) on the fluorescence signal of the sensor (∆F_Blue_/F_green_), indicating that the probes have a good selectivity for detection of Cu^2+^ (32.5 µM) in the presence of other metal ion interferes even at tenfold concentrations in drinking water.

To evaluate the sensitivity and selectivity of the designed probe in the presence of other interferences, the potential of interfering effects of other vitamins were tested. As shown in Fig. 6B, no apparent changes (< 3%) of fluorescence intensity ratio (F_blue_/F_green_) were found after the introduction of other vitamins (C, B_3_, B_5_) with concentration of 1.6 µM as interfering substances for selectivity studies of the probes for thiamine (0.16 µM). The investigation proved that the probe had good anti-interference properties for the detection of thiamine in real samples.

### Photostability and stability studies

The photostability of Zn/Cl-CQDs were investigated. The photostability was monitored by recording fluorescence spectra of Zn/Cl-CQDs under continuous UV irradiation at different time intervals. The results (Fig. S1) indicated that only ≤ 4% change in fluorescence intensity of the N-B CQDs were observed during 300 min. In addition, the Zn/Cl-CQDs solution has good stability (≤ 6% change in fluorescence intensity) and can be preserved away from light for at least 2 month at a refrigerator (4 °C).

Moreover, the stability of Zn/Cl-CQDs was studied by investigating the influences of pH (3- 11), and concentration of NaCl (0.1–1 M) (Fig. S2). As shown in Fig. S2A, the fluorescence intensities were decreased at the pH from 7 to 11 for both Zn/Cl-CQDs and riboflavin. Whereas the ratio of fluorescence intensities (F_green_/F_blue_) exhibited negligible changes (Fig. S2B). Therefore, stability of Zn/Cl-CQDs was almost independent on the pH values and could be applied at a broad pH range. But in these studies, pH 7 was chosen as optimum for further experiment. Moreover, the stability of the Zn/Cl-CQDs under various ionic (0.1–1 mol/L NaCl) strengths was also investigated (Fig. S3), and it was found that the fluorescence intensity of Zn/Cl-CQDs has no obvious changes.

### Real samples multi-component detection

In order to evaluate the practical applicability of the designed probe for sequential determination of riboflavin, Cu^2+^ ion and thiamine, VB_2_ tablet, drinking water and VB_1_ tablet were used as real samples, respectively. Table 2 indicates spiked and recovery results obtained by the ratiometric fluorescent probes for sequential multi-component detection of analytes in VB_2_ tablet, drinking water and VB_1_ tablet. In the real samples, the recovery results were in the range 93.14–98.44% with RSD between 1.65 and 2.84% (*n* = 3). The analytical results indicated a high level of agreement with their spiked value and label/manufacture’s claims.

**Table 2:** Analytical results for the sequential multi component detection of riboflavin, Cu^2+^ and thiamine in real samples (n = 3).

Sample	Spiked	Claimed content	Found	RSD (%, n = 3)	Recovery (%)
VB2 Tablet	–	470 nM	452.19 nM	2.84	96.21
	–	235 nM	218.88 nM	1.85	93.14
Drinking water (Cu^2+^)	0 μM	–	N.D	–	–
	16 μM	–	15.36 μM	1.43	96.01
	32 μM	–	31.44 μM	1.97	98.25
VB1 Tablet	–	6.63 μM	6.52 μM	1.65	98.44
	–	0.16 μM	0.15	2.17	93.38

## Conclusion

Sequential multi-component detection of riboflavin, Cu^2+^ ion and thiamine based on Zn/Cl-doped CQDs ratiometric fluorescent probes have special advantage than other fluorescent probes, because it can effectively eliminate or reduce background interferences thorough internal calibration and also provide the simultaneous detection of multiple analytes in different complicated matrices. Also, satisfying recovery results which were in agreement with their label/manufacture’s claims of VB_2_, VB_1_ tablets and spiked value of drinking water, proved the potential of designed platform for sequential multi-component detection of different analytes. Moreover, development of a metal-doped carbon dots such as novel Zn/Cl-CQDs, effectively adjusted their chemical reactivities and luminescence properties and also exhibited superior nanozyme catalyst activity in oxidation of the non-fluorescent substrate of thiamine to produce fluorescent substrate of thiochrome. More importantly, a new sequential ratiometric probe based on nanozyme catalyst activity of Zn/Cl-CQDs was constructed for highly sensitive and selective fluorescent turn-on response toward thiamine detection. According to this property of Zn/Cl-CQDs, upon the addition of thiamine to the probe solution, the blue emission of Zn/Cl-CQDs gradually recovered due to the produced fluorescent substrate of thiamine named as thiochrome. Moreover, subsequently a new sequential “turn-on” strategy was designed for accurate and sensitive detection of thiamine. This is a new concept for performance of ratiometric assays demonstrated with a Zn/Cl-CQDs-based catalyst activity which provides a basis for multi-component analysis as a new nanosensor platform for the future studies. Considering easy operation and high accuracy of these fluorescent sensors, the metal-doped CQDs can be promising in monitoring various analytes in drugs, water quality and body fluids.

## Supplementary Information

Supplementary Figures.

## Data Availability

All data generated or analysed during this study are included in this published article [and its supplementary information files].
