# Supplementary material for: Catalytic nanozyme Zn/Cl-doped carbon quantum dots as ratiometric fluorescent probe for sequential on-off-on detection of riboflavin, Cu2+ and thiamine
Source: Sci Rep. 2022 Oct 31;12:18276. doi: 10.1038/s41598-022-23055-6 (PMC9622855; doi:10.1038/s41598-022-23055-6)
Supplement: Supplementary file 1 — Supplementary Figures. [file 41598_2022_23055_MOESM1_ESM.doc]

Electronic Supplementary Material

**Catalytic nanozyme Zn/Cl-doped carbon quantum dots as ratiometric fluorescent probe for sequential on-off-on detection of riboflavin, Cu^2+^ and thiamine**

Sahar Dadkhah^a^, Ali Mehdinia^b^ [[1]](#footnote-2)*, Ali Jabbari^a^, Ahmad Manbohi^b^

*^a^Department of Chemistry, Faculty of Science, K. N. Toosi University of Technology, Tehran, Iran*

*^b^Iranian National Institute for Oceanography and Atmospheric Science, Tehran, Iran*


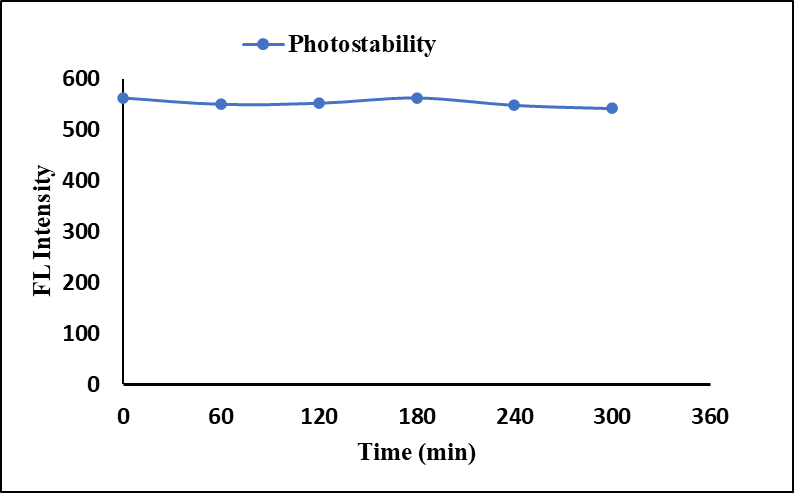


Fig. S1. The photostability of Zn/Cl-CQD under continuous UV irradiation at different time intervals


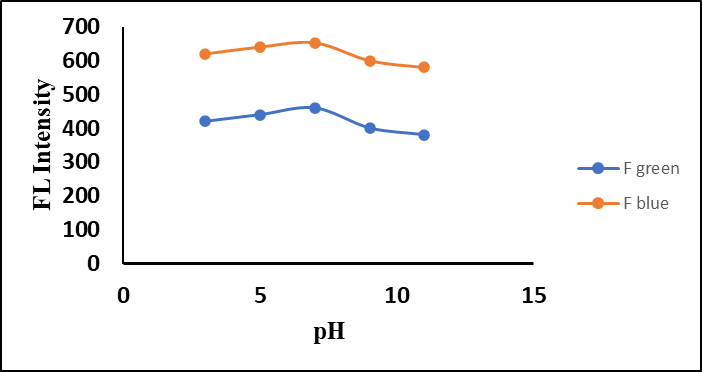


A


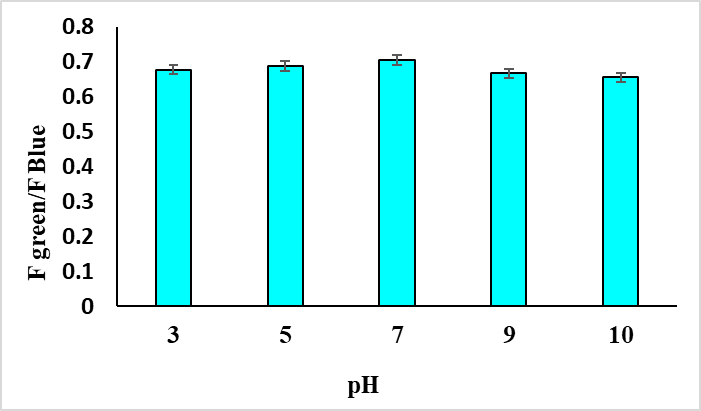


B

Fig. S2 . The stability of Zn/Cl-CQDs under various pH (3- 11). A) Effect of pH value on fluorescence intensities of Zn/Cl-CQDs (F _blue_) and riboflavin (F _green_).B) Effect of pH value on fluorescence intensities ratio of (F_green_/F_blue_)


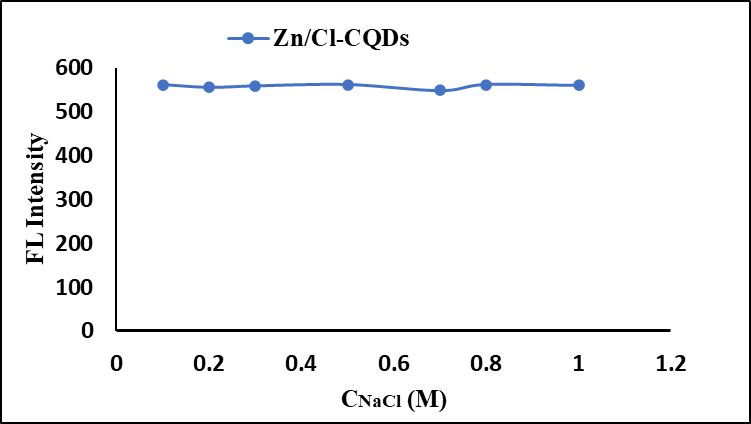


Fig. S3. Stability of the Zn/Cl-CQDs under various ionic (0.1-1 mol/L NaCl) strengths

**“The quantum yields”**

The fluorescence quantum yield of Zn/Cl-CQDs (in water) was measuring by the PL intensity in aqueous dispersion against quinine sulfate as a reference with a quantum yield of 54%.

The fluorescence quantum yield was calculated according to the following equation:

*Φ*_Sam_ = *Φ*_ST_ (*K*_x_ / *K*_st_)(*η*_x_ / *η*_st_)^2^ (1)

where Φ is the quantum yield, K is the slope from the plot of integrated fluorescence intensity vs absorbance, and η is the refractive index. The subscript “St” refers to the standard and “Sam” refers to the QDs.

The obtained Zn/Cl-CQDs exhibited excellent blue fluorescence (emission at 440 nm) and the fluorescence quantum yield were determined to be 33.8%.

*[1] Sun, D., Ban, R., Zhang, P. H., Wu, G. H., Zhang, J. R., & Zhu, J. J. (2013). Hair fiber as a precursor for synthesizing of sulfur-and nitrogen-co-doped carbon dots with tunable luminescence properties. Carbon, 64, 424-434.*

**“Catalyst activity study”**

The catalyst activity of the synthesized Zn/Cl-CQDs were further evaluated by degradation of thiamine to thiochrome in the absence and presence of Zn/Cl-CQDs followed by HPLC-separation and fluorescence detection.

The degradation of thiamine solution under different conditions was shown in Fig.S4. The negligible degradation was observed in thiamine individually and thiamine in the prescence of Cu^2+^. As observed from the Fig.S4, 70% of thiamine concentration were degraded after 5 minutes incubation in the presence of Zn/Cl-CQDs as catalyst platform.


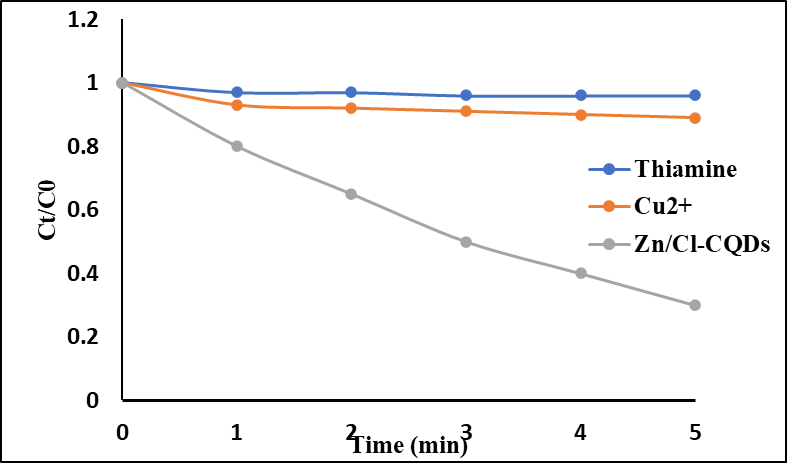


Fig.S4. The degradation of thiamine under different conditions

1. ^^ Corresponding author: Tel: +98 21 66944873; Fax: +98 66944869.

   E-mail addresses: [mehdinia@inio.ac.ir](mailto:mehdinia@inio.ac.ir) (A. Mehdinia) [↑](#footnote-ref-2)
